# Postoperative neurological aggravation after anesthesia with sevoflurane in a patient with xeroderma pigmentosum: a case report

**DOI:** 10.1186/1752-1947-7-73

**Published:** 2013-03-14

**Authors:** Salaheddine Fjouji, Mustapha Bensghir, Bahija Yafat, Najib Bouhabba, Elhoucine Boutayeb, Hicham Azendour, Nordine Drissi Kamili

**Affiliations:** 1 Department of Anaesthesiology, Military Hospital Med V Rabat, University of Med V Souissi, Rabat, Morocco

**Keywords:** Xeroderma pigmentosum, General anesthesia, Sevoflurane, Neurological worsening

## Abstract

**Introduction:**

Xeroderma pigmentosum is a rare autosomal recessive disease that causes changes in skin pigmentation, precancerous lesions and neurological abnormalities. It is a defect in the nucleotide excision repair mechanism. It has been reported that volatile anesthetics has a possible genotoxic side effect and deranged nucleotide excision repair in cells obtained from a patient with xeroderma pigmentosum.

We report an unusual case of postoperative neurological aggravation in a patient with xeroderma pigmentosum anesthetized with sevoflurane.

**Case presentation:**

A 24-year-old African woman, who has had xeroderma pigmentosum since childhood, was admitted to our hospital for a femoral neck fracture. A preoperative physical examination revealed that she had a resting tremor with ataxia. She had cutaneous lesions such as keratosis and hyperpigmentation on her face and both hands. There was no major alteration of cognitive function, muscular strength was maintained and her osteotendinous reflexes were preserved. Surgical fixation was performed under general anesthesia after the failure of spinal anesthesia. All parameters were stable during surgery. When she woke up four hours later, the patient presented with confusion and psychomotor agitation, sharpened reflexes and the Babinski reflex was present. Her postoperative test results and a magnetic resonance imaging scan were unremarkable. It was suggested that sevoflurane had had a probable deleterious effect on the neurological status of this patient.

**Conclusion:**

The anesthetizing of a patient with xeroderma pigmentosum is associated with a risk of worsening neurological disorders. At present, there are no clear recommendations to avoid the use of volatile agents in the anesthetic management of patients with xeroderma pigmentosum. More clinical and experimental research is needed to confirm the sensitivity of patients with xeroderma pigmentosum to sevoflurane and other halogenated anesthetics.

## Introduction

Xeroderma pigmentosum (XP) is a rare autosomal recessive disease, which is characterized by hypersensitivity of the skin to ultraviolet (UV) radiation and progressive neurological complications. It is caused by a defect in the nucleotide excision repair mechanism [1]. Estimated incidences vary from 1 in 20,000 in Japan to 1 in 250,000 in the USA, and approximately 2.3 per 1 million live births in Western Europe. Anecdotally, the incidence in North Africa and the Middle East is substantially higher [2]. Little information exists on the optimal anesthetic management of these patients [3-6]. It has been reported that volatile anesthetics have a genotoxic side effect in the cells of patients with XP and may worsen the symptoms [5,7]. However, up to now, there are no human data for this effect [8]. This report aimed at describing a postoperative neurological complication after general anesthesia in a patient with XP submitted to surgery for a femoral neck fracture and discussing the probability of sevoflurane as the factor of deterioration.

## Case presentation

A 24-year-old African woman, who had had XP since childhood, was admitted to our hospital for a femoral neck fracture after a fall on the stairs. Surgical fixation was indicated. A preoperative evaluation could not determine the genetic type of her XP. She had minor cutaneous forms on her face and on her hands, and had no history of tumor surgery. A neurological examination showed a minor decline in intelligence quotient (IQ), and an end tremor of the extremities associated with ataxic gait. She was calm, cooperative and oriented in time and space, with preserved muscle strength and deep tendon reflexes with no anomalies. A preoperative magnetic resonance imaging (MRI) scan did not show any sign of brain abnormalities. Her cardiovascular and pulmonary evaluation and biological test results were normal. The patient was premedicated with oral hydroxizine (50mg the day before and 50mg the morning of surgery). In the operating room, we initiated standard monitoring including pulse oximetry (SpO2), precordial cardioscopy, and noninvasive blood pressure (NIBP). A 16G peripheral venous catheter was secured and 1g cefazolin was administered. The patient was placed in a sitting position for the spinal anesthesia, and an injection of hyperbaric bupivacaine (12.5mg) and fentanyl (25μg) was given using a 25G Tuohy needle after checking the flow of cerebrospinal fluid. However, the motor and sensory blocks were insufficient. The patient’s Bromage score was 4 and her reaction to pinprick was significant. We converted to a general anesthesia induced by propofol 150mg, fentanyl 250μg, and cisatracurium 8mg and maintained by 2% sevoflurane with a 50% oxygen 50% air mixture. The procedure lasted 80 minutes without incident. The patient’s NIBP reading was between 136 and 92 as maxima, 81 and 56mm Hg as minima, her heart rate was 85 to 105 beats/minute and her SpO2 was between 98 and 100%. Postoperative analgesia was assured by paracetamol (1g), nefopam (20mg) and parecoxib (40mg). In the postoperative recovery room, the patient remained intubated and ventilated. Her unexplained nonawakening after about one hour induced us to monitor her postoperative residual curarization and to administer neostigmine. Her motor response was satisfactory. However, she did not wake up nor return to spontaneous ventilation after two hours, which induced us to perform a biological assessment (sodium, urea, glucose) that showed no abnormalities. After three hours, we noticed signs of awakening and spontaneous breathing. Her tidal volume was satisfactory. The patient became agitated, with abnormal movements that persisted despite the extubation, optimization of analgesia and a psychological reassurance. On examination, signs of pyramidal irritation appeared, such as sharpened reflexes, and the Babinski reflex was present, which necessitated transferring the patient to the MRI room for a scan; the images did not show any lesions that could explain these symptoms. Her agitation did reduce but other neurological disorders persisted for more than three postoperative days, with the appearance of memory disorders like false recognition and confusion in time and space. They were attributed to a neurological worsening of XP. Postoperative follow-up was recommended by neurologists. After three months, she is still presenting with confusion and cognitive decline, with memory disorders, ataxia and spasticity.

## Discussion

XP is a rare autosomal recessive disease, which is characterized by hypersensitivity of the skin to ultraviolet (UV) radiation and progressive neurological complications. It is mainly characterized by dermatological manifestations such as atrophy, keratosis, telangiectasis, hyperpigmentation and neoplasias in areas exposed to sunlight. The ophthalmologic symptoms are photophobia, corneal lesions, bilateral cataracts and a higher risk for benign and malignant eye tumors. There are eight genetic types of XP [9]; seven different genes involved in classical XP (XPA to XPG) and one gene for XP variant. Only patients with XPC, XPE and XP variant do not have neurological disorders, while the others show major and progressive neurological abnormalities. In this report, the genetic type of XP was not known, but it was probably one of those associated with neurodegenerative symptoms. Neurological abnormalities are found in 18% of XP patients [9]. The most common are mental retardation, spasticity, ataxia, microcephaly and peripheral neuropathy. Before the surgery, our patient had a minor decline of IQ, tremor of the extremities and ataxia. After surgery she appeared to develop irreversible cognitive decline, confusion, agitation and a pyramidal syndrome. Bone fragility in XP patients is probably due to a vitamin D deficiency related to patients’ protection from sunlight.

Little information exists on the optimal anesthetic management of patients with XP. For this type of surgery, a spinal anesthesia was chosen initially to prevent any neurological repercussions that could be caused by general anesthesia. After the failure of this technique, the available alternative was a general anesthesia induced by propofol, fentanyl, and cisatracurium and maintained by sevoflurane. There was no event that could explain the irreversible postoperative neurological degradation in our case. There was no hemodynamic instability, no transient hypoxia, and no electrolytic disorder was found. In the recovery room, residual curarization was eliminated by monitoring and neostigmine administration. A postoperative MRI scan did not show any cerebral lesions. Neurological disorders occurred in our patient, which could be attributed to the worsening of her XP symptoms by peri-operative factors including anesthetic agents.

The contribution of general anesthesia/anesthetics to postoperative cognitive decline (POCD) as a clinical entity has not been established, because patients under general anesthesia do not have an increased rate of POCD compared with patients under regional anesthesia [8]. But, a recent pilot study indicates that patients under isoflurane anesthesia may have a higher rate of POCD than patients under desflurane anesthesia after lower extremity or abdominal surgery [10]. In this case, surgical factors appeared to be controlled, and neurotoxicity of the medication is probably the most likely etiology.

Beyond that, it is not possible to separate the inhalational anesthetic effects from those of other anesthetic agents. Some evidence suggests that these unwanted effects vary according to each hypnotic’s specific pharmacodynamic and pharmacokinetic characteristics and its interaction with the individual patient [11]. Propofol reduces cerebral blood flow and cerebral O2 metabolism to a similar extent, has favorable pharmacokinetics and a high-quality recovery profile despite prolonged infusion [12,13]. Fentanyl is a half-life opioid analgesic. Its primary actions of therapeutic value are analgesia and sedation with a favorable side-effect profile and it may increase the patient’s tolerance for pain [14,15].

Data on volatile anesthetics-induced neurotoxicity in animal studies are accumulating rapidly [8]. However, there are, as yet, no human data for this effect. There has been no prospective randomized clinical trial to evaluate volatile anesthetics-induced neuroprotection or neurotoxicity [8]. For XP patients, Masuda *et al*. [5] recommend avoiding inhalation anesthetics and using total intravenous anesthesia (TIVA), based on a report by Reitz and Lanz [7]. These authors showed deoxyribonucleic acid (DNA) strand breaks and irreversible DNA exchange in the lymphocytes of two patients with XP after *in vitro* exposure to halothane and speculated about a possible genotoxic side effect of this drug. Karabiyik *et al*. [16] also showed transitory DNA damage by sevoflurane in human lymphocytes similar to isoflurane studied *in vivo.*

Associated with anesthetics-induced cell injury and death in neonatal animals, volatile anesthetics have also been shown to impair the cognitive functions of these animals [8]. There is, however, a lack of evidence for the causal relationship between the cell injury/death in the brain and the cognitive impairment in the animals after anesthetic exposure, both in the relationship between the genetic disorder in XP neuronal cells and the mechanism of sevoflurane-induced neurotoxicity.

On the other hand, volatile anesthetics have been used in clinical practice for nearly 160 years and are still the most commonly used anesthetics worldwide. Sevoflurane is often chosen for such patients based on its favorable properties for pediatric anesthesia, its ease of use, its malleability, and its clinical safety reported during its use even at high concentrations in inhalational anesthesia using a mask [4]. In our institution, sevoflurane has been used since 2006 with approximately 30 operations per day. Its safety is demonstrated but our experience with XP patients is still poor.

In clinical practice, among XP patients submitted to general anesthesia, Miyazaki *et al.*[6] reported a case of transient worsening of the neurological symptoms after anesthesia with volatile agents in previous surgery, whereas the intraoperative management and the postoperative course were uneventful with TIVA. Because the neurological deterioration in our patient appears to be probably related to the deleterious effects of anesthetics and sevoflurane is probably the most likely etiology because of its potential genotoxicity, we would suggest more experimental and clinical studies to affirm and explain a probable sensitivity of the neurodegenerative form of XP to volatile anesthetics. In the meantime, a preoperative neurological assessment is especially necessary in patients with the neurodegenerative genetic type XP, and using the TIVA option is preferable.

## Conclusion

The anesthesia of a patient with XP is associated with a risk of worsening neurological disorders. Because of their genotoxicity, based on experimental studies and case reports, the use of volatile anesthetics may have an adverse effect that has not yet been confirmed. At present, there are no clear recommendations to avoid volatile agents in the anesthetic management of patients with XP. More clinical and experimental research is needed to confirm the sensitivity of XP patients to sevoflurane and other halogenated anesthetics.

## Consent

Written informed consent was obtained from the patient’s next-of-kin for publication of this manuscript and accompanying images. A copy of the written consent is available for review by the Editor-in-Chief of this journal.

## Abbreviations

MRI: Magnetic resonance imaging; NER: Nucleotide excision repair; NIBP: Noninvasive blood pressure; POCD: Postoperative cognitive decline; SpO2: Pulse oximetry; TIVA: Total intravenous anesthesia; UV: Ultraviolet; XP: Xeroderma pigmentosum.

## Competing interests

The authors declare that they have no competing interests.

## Authors’ contributions

SF, MB, BY and NB analyzed and interpreted the patient data. SF, MB and EB were major contributors in writing the manuscript. HA and NDK made the final corrections. All authors read and approved the final manuscript.
